# Barriers to Infant and Child-feeding Practices: A Qualitative Study of Primary Caregivers in Rural Uganda

**Published:** 2015-03

**Authors:** Joyce Nankumbi, Joshua K. Muliira

**Affiliations:** ^1^Department of Nursing, College of Health Sciences, Makerere University, PO Box 7072, Kampala, Uganda; ^2^College of Nursing, Sultan Qaboos University, PO Box 66 Al Khod, Muscat, Sultanate of Oman

**Keywords:** Feeding practices, Infant-feeding, Malnutrition, Primary caregivers, Qualitative methods, Rural area, Uganda

## Abstract

The purpose of this study was to explore the barriers to the use of appropriate infant and young child-feeding practices by primary caregivers living in a rural Ugandan district. A community-based qualitative design and focus group discussions were used for collecting data from primary caregivers of children aged 0 to 24 month(s). On an average, each of the four focus group discussions had 11 participants. The focus group discussions were conducted using a structured interview guide and were tape-recorded. The recorded data were later transcribed and analyzed using qualitative thematic analysis techniques. All the participants were females, and the majority had low levels of education and at least one child in the age-group of 0-24 month(s) in their household. The findings show that the main barriers to the use of appropriate infant and young child-feeding practices fall under four themes: caregiver's knowledge about breastfeeding, caregiver's knowledge about complimentary feeding, influence of culture custodians on the caregivers, and patterns and burden of other responsibilities the caregivers have in the household. The four categories of barriers imply that there are various missed opportunities to implement hospital and community-based interventions to improve infant and young child-feeding practices, which is one way of preventing malnutrition. Therefore, in rural areas of Uganda, the major factors responsible for the high prevalence of malnutrition among infants and children are still those related to knowledge, culture, and social status of the primary caregivers.

## INTRODUCTION

Worldwide, 6.9 million children below the age of five years died in 2011, and 33% of these deaths are linked to malnutrition (1). The number of children who die from malnutrition-related problems in developing regions of the world, such as sub-Saharan Africa, is more than those in developed regions (1,2). In Uganda, malnutrition is one of the major public-health problems affecting infants and children. The prevalence of malnutrition among Ugandan children below the age of five years is estimated at 40%, and most of the malnourished children live in rural households (3). In Uganda, like other developing countries, childhood malnutrition is compounded by other prevalent diseases, such as malaria, diarrhoea, and pneumonia and these synergistically impact child health, leading to lifelong effects or death (4–7).

Malnutrition in infants/children also leads to psychosocial problems, such as impaired mental and physical development, reduced educational achievement, increased morbidity, and more time and money spent on taking care of sick children due to frequent morbidity (8,9). Therefore, the sequelae and risks associated with malnutrition have significant implications for the wellbeing of the family and future quality of the population in a country where the affected infants/children live (10,11). To address the problem of malnutrition in infants/children, one has to address the contributing factors and causes.

Leading authorities, such as the World Bank and the World Disaster Report, have identified the core factors that contribute to malnutrition in infants/children to be: foods used; infant and young child-feeding practices; barriers faced by caregivers; and availability of support resources (12,13). The current study focused on the barriers that prevent primary caregivers living in rural areas from using appropriate infant and young child-feeding practices (IYCFP). The IYCFP that were addressed are those related to breastfeeding and complementary feeding of infants in the age-group of 0-24 month(s).

Primary caregivers play a key role in feeding of infants/children and ultimately in their nutritional outcomes (8,14). In Uganda, the responsibility of childcare and nurturing is left to the mother but because of social situations, such as maternal death, teenage pregnancy, and work responsibilities, some infants/children are cared for by other female caregivers (aunts, grandmothers, and others). Many times, the primary caregivers experience several barriers which prevent them from adopting appropriate IYCFP and ensuring good nutrition outcomes for the infant/child. The common factors that influence nutrition outcomes of infants/children include socioeconomic status of the family, literacy levels, influence of relatives, and access to safe nutrient-rich complementary foods (15–19).

Unfortunately, many years of research and policy initiatives in sub-Saharan Africa have not effectively curtailed the prevalence of infant/childhood malnutrition (20). Some of the reasons behind the persistence of malnutrition in infants/children could be our limited understanding of the barriers faced by caregivers in rural areas where the majority of affected infants and children live. The purpose of this study was to explore the barriers to the use of appropriate IYCFP by primary caregivers of infants aged 0-24 month(s) living in a rural Ugandan district.

## MATERIALS AND METHODS

We conducted community-based focus group discussions (FGD) with primary caregivers to gain a comprehensive understanding of barriers to the use of appropriate IYCFP. Primary caregiver in this study is defined as any adult female person who is responsible for the day-to-day care and wellbeing of an infant/child between the age of 0 and 24 month(s), including biological mothers, grandparents, aunts, and others in cases where the biological parents are deceased or unavailable. A qualitative descriptive approach was adopted because it was found to be most suitable in generating a comprehensive initial understanding of the barriers to IYCFP in rural settings. The data used for this paper were collected as part of a bigger study whose results have been published elsewhere (21). The study was conducted in Luweero district, a rural area in central Uganda. Luweero district is one of the areas in Uganda, with a high rate of infant and childhood malnutrition. The population living in Luweero district is predominantly engaged in small-scale agricultural farming. The infant mortality rate (112/100,000 livebirths), stunting rate (36%), underweight rate (29%), and acute malnutrition rate (7%) in Luweero district are among the highest in central Uganda where the district is located. The prevalence of childhood malnutrition in central Uganda region is estimated at 5.3% (3).

The FGDs were conducted in the community where the participants lived. The participants were purposefully selected. For a person to be recruited to participate in the FGD, she had to meet the following inclusion criteria: currently or in the past 2 years was a primary caregiver to an infant/child of age between 0 and 24 month(s), had been a resident in the district for the past 2 years, and willing to participate in an FGD with other community members.

### Study procedures

The researchers approached the local council leaders of four selected villages to initiate the data-collection process. The four villages were selected because they had the highest population of infants/children in the age-range of 0-24 month(s) according to the information obtained from the district administrators at the time of data collection. The village leaders who are the custodians of the public records about residents in the village (the local council register of households) provided researchers with information about households with infants/children in the age-range of 0-24 month(s).

The homes that were approached were purposefully selected [only those identified as having infants aged 0-24 month(s)] and whose occupants were available during visits, which were used in order to publicize the study. The primary caregivers who consented to participate in the study were invited on a scheduled day to participate in an FGD at a central location in the village. A total of four FGDs were conducted and comprised 12, 10, 12, and 11 participants. One FGD was conducted in each of the four villages. Having participants in each FGD, who were from the same village and familiar with one another, helped facilitate open discussions.

The FGD on each day was guided by one of the researchers, using the interview guide. The proceedings of the FGD were recorded using a voice tape-recorder, and detailed notes were taken by the research assistants. The items on the FGD interview guide focused on: infant and young child-feeding practices, challenges faced when feeding infants and young children, and barriers to implementation of appropriate infant and young child-feeding practices. The FGDs were conducted in the local language (Luganda). The FGD data recorded in Luganda were later transcribed into written data in the same language. The transcribed data were then translated into English and back-translated into Luganda by two professional translators working independently to ensure accuracy and consistency before analysis. The fieldnotes were recorded in English because the research assistants were all bilingual and helped validate the transcripts.

### Ethical issues and approval

Ethical review and approval was obtained from the Uganda National Council of Science and Technology. The Uganda National Council of Science and Technology and Luweero district administrators gave permission to conduct the study. Informed written consent was taken from all the participants after full explanation of the nature, purpose, and procedures used in the study. The participants were informed that individuals’ names or names of the local villages will not be recorded or used and that they were free to withdraw from the study anytime. The location where the FGDs were held was booked to have only the investigators and participants at the time of data collection, and this helped maintain privacy. The participants were also informed that they will not be paid or given anything for participating in the study. Confidentiality was maintained throughout the study period (during data collection, data analysis, and reporting of the findings) by assigning the focus group a serial number. No other information of the participants that could identify the person was recorded during the study.

### Data analysis

To develop coding categories, the investigators independently read through the first FGD interview transcript translated into English, taking notes on the major topics discussed. The investigators then met to compare notes and revise individually-generated categories and to determine final coding categories. From this record, a codebook was developed to ensure consistent application of the final coding categories. The codebook included the criteria for applying each category and a brief excerpt of data exemplifying each category. Two coders trained in qualitative methods independently read and manually analyzed all the transcripts, using the codebook to identify similar categories. The codes were later reviewed by both researchers to ensure agreement and consistence of meaning in situations where differences arose. The agreed-upon codes were synthesized and grouped into exhaustive categories. The categories were then merged into themes representing the most common barriers that emerged from the FGD. The results presented below are organized in a way that shows themes explaining the main barriers, which prevent primary caregivers from using appropriate IYCFP.

## RESULTS

The results presented in Table 1 show the social and demographic characteristics of the 45 primary caregivers who participated in the FGDs. All primary caregivers were female and had at least one child in the age-group of 0-24 month(s). The majority of the primary caregivers reported that they were: biological mothers to the infant/child aged 0 to 24 month(s) in their households (87%); employed as housewife or farmer (94%), and had low levels of education (93%).

### Barriers to appropriate breastfeeding practices

There were four themes generated from the FGD data that explain the barriers to the use of appropriate IYCFP. The themes were primary caregivers’ knowledge about breastfeeding, primary caregivers’ knowledge about complementary feeding, influence of culture custodians, and pattern and burden of other responsibilities. The lack of knowledge was the predominant theme, and knowledge deficiency was also apparent in other aspects of childcare because most participants believed that monitoring what the infant or child eats should start when the child has started eating solid foods, when a child is sick, or when a child is undereating or overeating. The four themes that emerged from the qualitative data analysis are explained in details below.

#### Knowledge about breastfeeding

The participants reported inappropriate breastfeeding practices, and these were mostly stemming from lack of knowledge. The participants had low knowledge levels about components of breastfeeding, such as initiation of breastfeeding immediately after delivery, frequency of breastfeeding, and when to stop breastfeeding. Some of the statements from the FGDs highlighted below exemplify lack of knowledge about breastfeeding:

**Table 1.** Demographic characteristics of FGD participants

Characteristics	Category	Frequency (N=45)	Percentage
Age of primary caregivers (completed years)	18-24	10	22.2
	25-29	12	26.7
	30-34	13	28.9
	≥35	10	22.2
Relationship to the infant or child	Biological mother	39	86.7
	Grandmother	6	13.3
Religion	Christian	32	71.1
	Others	13	28.9
Marital status	Married	41	91.1
	Single	4	8.9
Level of education	No formal education	23	51.1
	Primary education	19	42.2
	Secondary and above	3	6.7
Occupation status	Housewife	16	35.6
	Farmer	26	57.8
	Others	3	6.7

**FGD 1:** “Midwives always tell us to put the baby on the breast after delivery but this does not help because the milk is absent on the first day, and this is very challenging.” “I would end up giving my baby tea to satisfy the baby; besides, this also helps to clean the baby's intestines and to prevent baby's abdominal pains.”
**FGD 2:** “My child was born with flu, I had to give and I gave her passion fruit juice.”
**FGD 3:** “In most cases, I have no milk immediately after childbirth, so what I do, I start the child on water with sugar or glucose water until the milk starts coming. This also helps me to stop the problems of breast-pain during sucking.”
**FGD 4:** “I stopped breastfeeding my children at 3 months because usually I do not produce enough milk to breastfeed the baby up to 6 months.”

#### Knowledge about complementary feeding

The participants mentioned that weaning off infants using other feeds should be started immediately after birth, and this was one of the indicators of lack of knowledge about complementary feeding. Other participants mentioned that weaning off a child was started at 3 months, although they had heard that weaning should start at 6 months. The reasons given for early weaning were chronic lack of breastmilk to continue breastfeeding the baby up to 6 months and constant crying of the child because of hunger and, therefore, the need to give satisfying foods other than breastmilk. Porridges were the commonly-used complementary feeds. The porridges commonly used were those made from cassava-flour, maize or corn-flour and millet-flour. The participants also reported that they introduced foods, such as rice, Irish potatoes, sweet potatoes, and cooked green banana (*Matooke*), which they believe are soft and suitable to meet the infant's/child's nutritional needs. The following statements from the FGDs capture some of the points, which show lack of knowledge about complementary feeding:

**FGD 1:** “I started giving porridge to my baby at 2 weeks after birth because I did not have enough milk, and, at the same time, I did not have sugar to add to the baby's porridge. This works very well on all children I have heard before.”
**FGD 2:** “I introduce the other foods at 3 months because the milk is no longer enough for the baby, this you can tell when the child overcries meaning that they do not get satisfied. The child likes the food because, at the same time, the child licks her/himself when you make them taste when I am eating my own food.”
**FGD 3:** “I feed my child on *Matooke*, rice, and Irish potatoes; they are the soft foods that a child can easily chew and digest. At that time, the child's intestine cannot handle the other kinds of foods. These foods help him grow.”
**FGD 4:** “At four months, the child wants to eat; they grab food when you are eating your own food. I am tempted to start giving food to the child. I gave food, and she ate all; whatever I eat she eats.”

#### Influence of culture custodians

Cultural practices resulting from the influence of respected members of the community or family also stood out as a significant barrier to the use of appropriate infant and young child-feeding practices. The cultural practices seem to be entrenched by elders in the community or highly respected people in the family, and this led us to generate the theme “influence of culture custodians” as a barrier to the use of appropriate IYCFP. The statements below show the genesis of the theme:

**FGD 1:** “My mother was there when I gave birth, and she told me that the only way to prevent colic pain and keep my baby healthy was to give my baby juice squeezed from a tomato and also to take my baby to the ancestral home with other relatives to see the baby.”
**FGD 2:** “You see these women here; they do what the mother who gave birth to them does. I feed only foods which I know are our tribes’ foods. My mother in-law will think I am not taking care of children, if I don't start giving them food at an early age after birth.”
**FGD 3:** “When I spend more than two nights away from the baby, the breastmilk becomes stale, and you cannot give it to the baby …how can I start expressing breastmilk? It is a taboo.”
**FDG 4:** “As you know, as a woman you go to the mother in-law and older women to show you how to feed the man and child. Sometimes, these sons have to be raised strong, and we give them solid food soon to help them become strong people.”

#### Pattern and burden of other responsibilities

Some primary caregivers reported that they have heard before that a child can be exclusively breastfed for 6 months by the mother or need to be fed well on time. However, this report was followed by the complaints of lack of time because of work and other responsibilities in all FGDs. Because of lack of time, they could not exclusively breastfeed or give their children adequate food on time. In all the FGDs, the burden of other responsibilities was reported to be one of the major reasons for inadequate duration of breastfeeding or complementary feeding. Most participants reported that they give food to their children 2 to 3 times a day but the frequency varied and depended on the other activities and responsibilities the caregiver had on a given day. The participants stated that they feed their children after cooking, and the food is served on the child's own plate, especially if the child is aged more than 8 months. This is done to catch up with other responsibilities and work. Sometimes, the caregivers supervise and monitor the child's feeding, especially if they were also having a meal at the same time. The statements from the FGDs presented below exemplify the significance of the barrier of pattern and burden of other responsibilities primary caregivers have to perform:

**FGD 1:** “I exclusively breastfeed my children for 3 months because the milk is not always enough, and I have so many things on my head to finish in the garden, the house, and other children. So, I always stop early because the family need soap and fuel.”
**FGD 1:** “When a child is one year, they can be served on their plates and are able to feed themselves or you request the older child to help feed the child because you may not be at home, you could be in the garden. This is what we do down here.”
**FGD 2:** “When I leave for the garden, the child can stay with his or her siblings. When I come back I have to sit down to breastfeed and once they start to play while breastfeeding, then I stop because it's me the same person who has to do most of the work, I have to go to fetch water and make a meal for everybody and, sometimes, you have to do laundry as well.”
**FGD 2:** “Due to the heavy workload we have as women, our homes have only two meals in a day. And as long as the child is above 8 months, then they are old enough to survive but also if I see that my child is small or is not growing well I will be keeping some food for him which I can give separate from the meal times.”
**FGD 3:** “Most of the times, you have a newborn baby and another child. Even if less than 2 years, I feel they can survive on the adult meals. A child of 1 year is big enough to learn how to feed him or herself, I won't have the time to do that.”
**FGD 4:** “I do secure the food and, to some extent, the income of my family; I am able to go with the child to search for firewood and palm leaves to make mats for sale.”
**FGD 4:** “Sometimes, I am too busy and find my child still eating. I have to stop her from eating too much. She finishes eating her regular meal, and she wants more food.”

### Missed opportunities to enhance IYCFP

Our analysis shows that there are several opportunities that can be tapped to enhance appropriate breastfeeding practices for infants and children in rural areas. Table 2 presents a summary of some of the missed opportunities to enhance appropriate IYCFP-related breastfeeding. Interventions, such as health education, nutrition navigators, involvement of cultural leaders, and peer support groups, can be used for enhancing primary caregivers’ knowledge about breastfeeding in antenatal clinics, postnatal clinics, child immunization clinics, and community outreach programmes. Such opportunities can help provide correct information about recommended breastfeeding practices and support to primary caregivers and men in families to implement appropriate IYCFP. Other interventions, such as using expressed breastmilk for child-feeding while the mother is away to maintain exclusive breastfeeding and group-farming to join labour, can help ensure adequate quality nutrition and reduced amount of time spent in agricultural activities, such as planting and harvesting by one family member.

In Table 3, we summarize some of the missed opportunities that can be taken advantage of to enhance the use of appropriate IYCFP relating to complementary feeding. Some of the interventions that can help overcome the barriers of knowledge and burden of work and responsibilities and promote appropriate practices relating to complimentary feeding include: fortification of local foods and availing them in easy-to-use packets that can be given by the fathers, siblings, and other members of the family when the primary caregiver is away; training of local people in the production of ready-to-use fortified food for children; community health education about complimentary feeding; community nutrition navigators and counsellor to support primary caregivers and men's involvement in infant-feeding; and introduction of varieties of crops yielding high nutrition. The trained personnel can also serve nutrition extension workers to bring the services of nutritional education, counselling, and support closer to where primary caregivers and families live in rural areas.

## DISCUSSION

The results of the present study show that knowledge, influence of culture custodians, and patterns and burden of work and other responsibilities at home are the main barriers affecting the use of appropriate infant and young child-feeding practices (IYCFP). The primary caregivers lacked knowledge about both recommended breastfeeding and complimentary feeding practices. Lack of knowledge about aspects, such as appropriate complimentary feeding and foods (quality and quantity), has been reported in other developing countries (22). Caregiver's lack of knowledge has also been found to be associated with spending insufficient time to feed the child and insufficient consumption of adequate amounts of complementary foods to meet the child's energy and micronutrient needs (22).

The primary caregivers were following recommendations and information given by influential members in the family or community whom they respected because of their perceived wisdom about cultural issues, traditions, and health matters to guide their infant and child-feeding practices. This influence, advice, and wisdom from influential family or community members, such as mother-in-laws, grandparents, and others, led us to the theme of influence of culture custodians. The influence and impact of culture custodians, such as grandmothers on primary caregivers, has been reported in other sub-Saharan African countries, like Senegal (23). Therefore, in rural communities, individuals who are perceived to be a source of wisdom and guidance on cultural ways of life can play a critical role in shaping feeding practices and, subsequently, nutrition outcomes and child health (23).

Cultural practices sustained by recommendations and advice of family and community members are still one of the least-explored issues and were addressed by efforts to promote the use of appropriate IYCFP in developing countries. The challenge posed by the barrier of influence of culture custodians is unique and has to be addressed with care because individuals, such as grandmothers, are also, many times, caregivers themselves, have strong social networks and exercise significant collective influence on practices relating to pregnancy, behaviour of young women, and care of sick children (24).

The other major barrier to the use of appropriate IYCFP was the pattern and burden of other responsibilities the primary caregivers had to meet on a daily basis. The examples commonly listed by the participants were involvement in agriculture and household chores. The primary caregivers hinted on a very high burden of househod chores, and these tended to intensify during agricultural planting and harvesting seasons. The primary caregivers received limited to no support from the male adults (or husbands) in the family towards meeting the infant/child-feeding needs or reducing the burden of household chores and other responsibilities. This finding is similar to what has been reported by other studies conducted in developing countries. For instance, a study conducted in the Bolivian Andes found that heavy agricultural workload, lack of support for child-feeding from spouses, and individual influences were major barriers to improving child-feeding practices (25). In the Bolivian Andes, low crop diversity was also found to be a major barrier (25).

**Table 2.** Examples of missed opportunities to overcome barriers to appropriate IYCFP-related to breastfeeding

Practice	Category of barrier	Examples of specific barriers	Examples of missed opportunities
Initiation of breastfeeding	Lack of knowledge Influence of culture custodians	Lack of knowledge about benefits of breastmilk and colostrum Lack of knowledge about correct placement of baby (latching) Lack of knowledge about advantages of early initiation of breastfeeding after childbirth Cultural practice of giving prelacteal feeds Influence from respected cultural and family elders	Health education about breastfeeding initiation Use of evidence-based intervention, such as ‘kangaroo care’ Implementation of mother rooming with child after delivery Teaching mothers in antenatal, postnatal and immunization clinics Using community professional and peer breastfeeding counsellor Community-based breastfeeding navigators Involvement of community leaders and the whole family in IYCFP initiatives Health education of community leaders about IYCFP Community-based infant-feeding professional and peer-counselling
Breastfeeding on demand	Pattern and burden of other responsibilities	Lack of time to breastfeed (agricultural work and other household chores) Exhaustion from household work leading to limited/poor breastmilk production Separation from child, especially due to work and lack of maternity leave in informal sector Caregiver cannot breastfeed (grandmother)	Community support groups for primary caregivers Group-farming to encourage joint labour Involvement of men and other family members in infant-feeding Community education and teaching about IYCFP Health education about the use of expressed breastmilk
Exclusive breastfeeding	Pattern and burden of other responsibilities	Failure to maintain adequate milk production due to lack of knowledge, rest, and exhaustion Heavy workload of the mothers	Health education about the use of expressed breastmilk Teaching regarding IYCFP role in sharing by family members Health education about nutrition for lactating mothers Health education about alternative to breastmilk for non-biological mothers
Continued breastfeeding	Lack of knowledge	Lack of knowledge about recommended breastfeeding practices Challenges of maintaining adequate breastmilk production	Teaching about the use of expressed breastmilk Strengthening family support systems to encourage primary caregivers to continue breastfeeding Community-based peer-counselling on infant-feeding

**Table 3.** Examples of missed opportunities to overcome barriers to appropriate IYCFP-related to complementary feeding

Practice	Category of barrier	Examples of specific barriers	Examples of missed opportunities
Prelacteal feeding	Lack of knowledge Influence of culture custodians Pattern and burden of other responsibilities	Cultural beliefs of cleansing the intestine to checking whether baby is alive	Delivering in hospital setting with access to lactation and child nutrition professional support Health education about IYCFP at health facilities Peer-counselling on infant-feeding and child nutrition navigators
Timing of introduction of complementary foods	Lack of knowledge Influence of culture custodians Pattern and burden of other responsibilities	Lack of knowledge about appropriate time to introduce complementary foods Advice by influential cultural elders about complementary feeding Lack of time to feed or monitor child-feeding	Accessibility to mass media, such as radio-based educational messages Communication channels, such as women's associations Community-based peer-counselling on infant-feeding Teaching of influential cultural leaders to change misconceptions
Frequency of using complementary feeds	Lack of knowledge Influence of culture custodians Pattern and burden of other responsibilities	Lack of knowledge about childhood nutritional requirements Lack of male participation in child-feeding	Introduction of high-yielding crops to increase home incomes Teaching and supporting males and other family members to be active participants in IYCFP Increasing access to pre-packed ready-to-use fortified complementary food to reduce time spent in preparing child's food
Types of complementary foods	Lack of knowledge Influence of culture custodians Pattern and burden of other responsibilities	Lack of information and awareness about best foods for young children Lack of access to fortified foods for infants Advice and recommendations to use only culturally-familiar food choices	Teaching the use of locally-available foods as complementary foods Increasing access to pre-packed fortified foods made using local crops Community-based training on making fortified foods for children Training in food preservation for use during agricultural off-seasons Introduce new highly-nutritious crops to grow for consumption or sale
Active feeding behaviours	Lack of knowledge Influence of culture custodians Pattern and burden of other responsibilities	Lack of knowledge about active child-feeding Cultural practices of allowing very young child to feed themselves Lack of participation by males and extended family members in child-feeding	Health education about active child-feeding and related advantages Encouraging positive cultural practices, such as *Nakawere* (rest from all work after childbirth) to rest and allow mother to spend time with child Community-based counselling on infant-feeding and support groups Increasing access to pre-packed ready-to-use fortified complimentary foods to reduce time spent in preparing child's food

The limited involvement of men in the day-to-day care of the child, child-feeding and nutrition activities is common in sub-Saharan Africa (24) and shows a potential opportunity that can be explored to reduce the burden of work responsibilities faced by primary caregivers. Encouraging and promoting male members of rural families to participate in IYCFP can also help enhance child's bonding with the father, in addition to addressing the barrier of patterns and burden of other responsibilities faced by primary caregivers. Lack of time to feed the child because of agricultural and other household chores and poverty are critical factors because other studies have shown that, even after implementing interventions, such as teaching and demonstrations of appropriate feeding practices, the two factors still curtail the caregivers’ ability to use appropriate IYCFP (26).

Barrier of the lack of knowledge is especially significant because it shows that, in a way, health professionals have so far been unable to proactively and effectively use the available opportunities to enhance primary caregivers’ ability to use appropriate IYCFP (27). Lack of knowledge may be impacting critical aspects of child-feeding, such as early initiation of breastfeeding, exclusive breastfeeding for six months, and timely introduction of age-appropriate complementary feeding, all of which are key aspects of the process of preventing childhood malnutrition and associated mortality (28,29).

Our critical analysis of the barriers led us to ponder about the missed opportunities to enhance appropriate IYCFP in rural areas. The missed opportunities, in our view, are the aspects which can easily be corrected or addressed by implementing interventions that promote IYCFP and reduce childhood malnutrition, using the existing, cheap or innovative strategies. The strategies that can be used include breastfeeding counselling; community-based peer-counselling on feeding (30,31), social groups to encourage and motivate mothers to achieve successful lactation (32), community and health facility-based health education, skilled counselling to provide accurate information about IYCFP, training of healthcare staff in IYCFP, using community nutrition extension workers and IYCFP navigators, and primary caregiver support groups (33). Such strategies can help overcome the barriers to the use of appropriate IYCFP and associated infant/childhood malnutrition in rural communities in a holistic way because these can easily focus on primary caregivers, men, extended family members, and the community.

### Strengths and limitations

This is a qualitative study, and the sample was not representative of the entire population of primary caregivers in the district and did not include men or a large number of grandmothers and other people in the community, who are regarded as key informants and source of advice on cultural issues relating to nutrition and infant-feeding. The district where the study was conducted is increasingly becoming multitribal and, therefore, it is difficult to generalize the findings without further studies to assess the beliefs of community members about IYCFP across various tribes. Despite the limitations of this study, the findings provide a starting point for serial surveys and interventions to reduce the impact of negative culture, lack of knowledge, and work burden on IYCFP and childhood nutrition outcomes.

### Conclusions

In rural areas, primary caregivers are unable to use appropriate IYCFP to enhance the nutritional status of their children because of lack of knowledge, lack of time, and influence from culture custodians. These barriers also imply that there are various opportunities that can be used in implementing hospital- and community-based interventions to improve infant and young child-feeding practices as a way of preventing malnutrition. The opportunities exit at the level of the family, community, and healthcare system.

## ACKNOWLEDGEMENTS

The study was funded by the Carnegie Foundation (Grant number: 8741)

**Conflict of interest:** Authors declare no conflicts of interests.
